# Taurine Alleviated the Negative Effects of an Oxidized Lipid Diet on Growth Performance, Antioxidant Properties, and Muscle Quality of the Common Carp (*Cyprinus carpio* L.)

**DOI:** 10.1155/2024/5205506

**Published:** 2024-06-21

**Authors:** Dan Liu, Jiali Mi, Xiao Yan, Chaobin Qin, Junli Wang, Guoxing Nie

**Affiliations:** ^1^ College of Life Science Henan Normal University, Xinxiang 453007, China; ^2^ Aquatic Animal Nutrition and Feed Research Team College of Fisheries Henan Normal University, Xinxiang 453007, China

## Abstract

In the present study, we conducted a 10-week culture experiment to investigate the effects of taurine on the growth performance, antioxidant properties, and muscle quality of the common carp fed an oxidized lipid diet. There were five experimental groups with three replicates each. Based on the fresh fish oil group (FO), equal amounts of oxidized fish oil (with a thiobarbituric acid-reactive substances value of 49.57 ± 2.34 mg/kg) and 0 g/kg (OFO), 4 g/kg (OT4), 8 g/kg (OT8), and 12 g/kg (OT12) taurine were added to the diet, while the same composition ratio was maintained by changing the microcrystalline cellulose content. Compared to the FO group, the feed conversion ratio, weight gain rate, muscle crude lipid, and n-3/n-6 polyunsaturated fatty acids (PUFA) ratio were significantly lower in the group OFO (*P* < 0.05). In addition, compared to the FO group, OFO fish showed an increased content of malondialdehyde and protein carbonylation and decreased hardness, brightness, pH, superoxide dismutase, and catalase levels in the muscle (*P* < 0.05). Notably, the growth index significantly improved in the OT4 group compared to that in the OFO group (*P* < 0.05). In addition, dietary taurine increased the crude lipid content, n-3/n-6 PUFA, antioxidant capacity, hardness, springiness, brightness, pH, and collagen content in the muscle compared with OFO fish (*P* < 0.05). Moreover, taurine supplementation significantly reduced myofiber diameter and increased myofiber density (*P* < 0.05) and enhanced the expression levels of paired box 7 (*pax7*), myogenic factor 5 (*myf5*), myogenic differentiation antigen (*myod*), and myogenic regulatory factor 4 (*mrf4*) compared with that of the OFO group (*P* < 0.05). Considering growth performance and muscle quality, the optimal supplemental levels of taurine in the oxidized lipid diet were 4 g/kg and 6.84–7.70 g/kg, respectively.

## 1. Introduction

Lipids are essential components of diet and most biological systems, providing animals with energy, essential fatty acids, and soluble vitamins, which are structural components of cell membranes [1]. However, lipids are easily oxidized through different pathways. The degree of oxidation depends on many intrinsic and extrinsic conditions, including the degree of fatty acid unsaturation, the composition of components, environmental factors, and transport and preservation techniques, so on [2]. Lipid oxidation is prevalent in fish feed, and harmful compounds such as alcohols, ketones, and aldehydes reduce the nutritional value of fish feed and damage the health of aquatic animals [3]. One study showed that oxidized lipids destroy the antioxidant system, induce apoptosis, and compromise hepatopancreatic mitochondrial function in largemouth bass (*Micropterus salmoides*) [4]. Studies on the rice field eel (*Monopterus albus*) have confirmed that oxidized fish oil elevates oxidative stress in muscles and increases muscle fiber loss caused by autophagy [5]. In addition, feeding blunt snout bream (*Megalobrama amblycephala*) oxidized lipids for 12 weeks resulted in growth inhibition, oxidative stress induction, and intestinal integrity disruption [6]. Therefore, the harmful effects of oxidized lipid diets on aquatic animals cannot be ignored, and there is an urgent need to find effective dietary strategies to enhance fish health and improve farming benefits.

Among the many ways to alleviate lipid oxidation, adding antioxidants that destroy or delay the oxidation chain reaction is the most effective, convenient, and economical method [2]. Taurine is a natural antioxidant that can scavenge peroxides and superoxides in vitro [7]. The antioxidant capacity of taurine has been demonstrated in several *in vivo* experiments. Studies in fish have shown that taurine can improve antioxidant capacity by reducing oxidative stress and increasing the activity of antioxidant enzymes (such as superoxide dismutase (SOD), catalase (CAT), and other antioxidant enzymes) [5, 8]. Previous studies have shown that adding taurine to feed improves the feed conversion ratio (FCR), feeding rate (FR), and growth performance of golden pompano (*Trachinotus ovatus*) [8] and juvenile turbot (*Scophthalmus maximus* L.) [9]. In addition to its antioxidant and growth-promoting effects, taurine exhibits various other physiological activities, including membrane stabilization, calcium homeostasis, and immune regulation [10]. Therefore, taurine has the potential to be used as a feed additive to alleviate the damage caused by oxidized lipid diets in fish.

The common carp (*Cyprinus carpio* L.) is an omnivorous freshwater fish with important economic and edible value, and its rich historical and cultural background can be traced back to 8,000 years ago [11]. Muscle is the most nutrient-dense and tasteful portion of fish. With advancements in human living standards, consumers are becoming increasingly concerned about fish muscle quality [12]. However, quality issues, such as soft muscle texture, bad flavor, and decreased nutritional value of farmed common carp, have substantially affected consumer acceptance in recent years [13]. Recent studies on omnivorous fish have shown that taurine positively affects muscle quality. A relevant study on the rice field eel (*M. albus*) found that taurine upregulated the expression levels of muscle development genes such as *myf5*, *myod*, and *mrf4*, reduced myofiber loss, and maintained muscle homeostasis [5]. In addition, studies on Nile tilapia (*Oreochromis niloticus*) have shown that taurine plays an important role in improving skin color and promoting muscle quality [14]. Nevertheless, research on the effects of taurine on fish muscle quality is limited, and the specific mechanisms need to be studied further.

## 2. Methods and Materials

### 2.1. Ethics Statement

All animal studies were conducted in strict accordance with the “Guidelines of Laboratory Animal Treatment and Usage” and are approved by the Committee for Institutional Animal Protection and Use of Henan Normal University (HNSD-2023 BS-1232).

### 2.2. Preparation of Oxidized Oils

Oxidized fish oil was prepared by inserting an air pump tube into fresh fish oil and continuously stirring the oil while incubating it in a water bath at 55°C for 5 days [15]. After oxidation, the peroxide values were measured in triplicate.

The peroxide value was determined according to the Chinese National Standard for Food Safety (GB/T 5009.37-2003): The sample was weighed (about 3 g) and dissolved in 30 mL mixed solution (trichloromethane and glacial acetic acid). Subsequently, 1 mL of saturated potassium iodide solution was added, and the reaction was carried out in the dark for 3 min. After adding 100 mL of water, the mixture was titrated against a standard solution of sodium thiosulfate using starch as an indicator. The peroxide value of fresh fish oil was 2.51 ± 0.11 meqO_2_/kg, whereas the value of oxidized fish oil was 175.39 ± 3.83 meqO_2_/kg [16, 17].

### 2.3. Experimental Diets

Five isonitrogenous and isolipidic experimental diets (FO, OFO, OT4, OT8, and OT12 groups, respectively) were configured, as shown in Table 1. Cottonseed, soybean, rapeseed, and fish meals were the main protein sources, whereas fish oil was the lipid source. The basal control diet (FO) was supplemented with 30 g/kg fresh fish oil, whereas the other diets contained equal amounts of oxidized fish oil and 0 g/kg (OFO), 4 g/kg (OT4), 8 g/kg (OT8), or 12 g/kg (OT12) taurine, the same composition ratio was maintained by changing the microcrystalline cellulose content. As taurine is abundant in animals but poor in plants [18], a higher plant-based diet was established in this study to reduce the influence of primitive taurine in the diet. The taurine content was determined using high-performance liquid chromatography (HPLC), as shown in Table 1. Considering that cellulose improves diet pellet durability and reduces pellet expansion [19], we added about 10% of cellulose to the diet without affecting the nutrient digestibility of the fish [20]. All plant ingredients were crushed to a fine powder with particle size <177 *μ*m, then thoroughly mixed with other fine ingredients and trace components, followed by lipids and distilled water (30%, w/w). The mixed ingredients were cold-extruded into pellets with a diameter of 2 mm and length of 3–4 mm using a pellet machine (South China University of Technology, Guangzhou, China). The material temperature was 27–30°C, and the moisture was 12.5%–13.5% during the pelleting process. Finally, the pellets were air-dried in the shade until the moisture content was less than 10% and stored in sealed vacuum bags at 4°C for later use.

### 2.4. The Rearing Trial

In this experiment, 225 healthy common carp (8.84 ± 0.02 g) were randomly assigned into 15 square cages (2 m × 2 m × 1.5 m) with 15 fish in each cage and divided into five treatment groups with three replicates in each group. The farming experiment was conducted at an experimental base (Luohe, Henan Province) consisting of an open fish pond (6,667 cm^2^) with three large oxygenators and a water-changing system. The experimental fish were acclimated on an FO diet for 2 weeks before the formal trial. During the experiment, all fish were fed three times a day (07:30, 11:30, and 17:30) at 3%–5% of their body weight, while feed intake was modified once a week for 10-weeks. The temperature (25.7 ± 0.70°C), pH (7.4 ± 0.2), ammonia nitrogen content (0.4 ± 0.2 mg/L), and dissolved oxygen (9.0 ± 1 mg/L) of the pond water were measured daily throughout the feeding period to meet the standards of farmed carp.

### 2.5. Sample Collection

After 24 hr of fasting, the fish were sedated with tricaine methanesulfonate (Aladdin, Shanghai, China), and the total number and weight of fish were recorded. Three carp were randomly chosen from each cage to measure body length, viscera, and hepatopancreas weights. Muscle blocks (2 cm × 1 cm × 1 cm) above the lateral line were placed in a 4% paraformaldehyde solution for morphological analysis. White muscles and hepatopancreas were immediately deposited in 1.5 mL centrifuge tubes containing no DNase or RNase, briefly frozen in liquid nitrogen, and preserved at −80°C for real-time PCR and biochemical detection. Muscles were gathered and kept at −20°C to determine approximate composition and physicochemical properties. Dorsal muscle color (brightness, redness, and yellowness) was measured using a spectrophotometer (3nh, Guangdong, China) after the fish were skinned.

### 2.6. Thiobarbituric Acid-Reactive Substances (TBARS)

The TBARS of fish oils and feeds were tested based on the principles of Sinnhuber (1977) and slightly modified according to the description of Xia (2009) [21, 22]. After reacting with 2-thiobarbituric acid (TBA), an equal volume of chloroform was added to the sample solution, mixed, and centrifuged at 3,000 rpm for 10 min. The absorbance of the supernatant was measured at 532 nm.

The TBARS was expressed as mg of malondialdehyde (MDA)/kg of the sample and calculated using the following equation:

(1) $$\begin{array}{c} \text{TBARS }\left(\text{mg}/\text{kg}\right)=\left(A_{532}/Ws\right)\times 9.48 \end{array}$$

where *A*_532_ is the absorbance (532 nm) of the supernatant, *Ws* is the weight of feed or fish oil (g), and “9.48” is a constant derived from the dilution factor and molar extinction coefficient (152,000 M^−1^cm^−1^) of the red TBA reaction product. All samples were tested in triplicates. The TBAR (in oil) of normal fish oil and oxidized fish oil were 32.73 ± 1.24 and 49.57 ± 2.34 mg/kg, respectively.

### 2.7. Antioxidant Enzyme Activities and Peroxide Levels

The contents of MDA, SOD, and CAT in the muscle and hepatopancreas, as well as the content of lactic acid in the muscle, were measured using commercial kits (Nanjing Jiancheng, Nanjing, China).

The muscle protein carbonylation (PCO) was measured using 2,4-dinitrophenylhydrazine (DNPH) spectrophotometry, as previously described [23]. The absorbance was determined at 370 nm.

### 2.8. Nutrient Composition and Muscle Texture Characteristics

The approximate composition of the muscle and feed was determined according to the AOAC [24] standard method. Briefly, samples were freeze-dried to constant weight at −50°C to detect moisture. The Kjeldahl method was used to measure crude protein content. The crude lipid content was determined by Soxhlet extraction using a fat analyzer (OPSIS, Skåne, Sweden).

The crude fiber content of the feed was determined according to the national standard (GB/T 6434-2022), as follows. The diet sample (approximately 1 g) was placed in a filter bag and boiled with acid (0.13 mol/L sulfuric acid) and alkali (0.23 mol/L potassium hydroxide solution) for 30 min. Then, the sample was degreased with acetone and dried to a constant weight at 105°C. The sample was then placed in a crucible of constant weight and carbonized in an electric furnace. Final ashing was performed in a muffle furnace (Shanghai Yiheng, Shanghai, China) at 600°C for 3 hr and weighed after cooling.

Muscle texture was tested using a slight modification of a previously described method [25]. Four muscle blocks from both sides of the fish were cut to the same thickness and size (1.0 cm × 1.0 cm × 0.5 cm) for texture analysis. The muscle samples on one side were steamed in boiling water for 5 min to analyze the texture of cooked muscle, while the other side was tested for the texture of raw muscle. A texture analyzer (Brookfield, Middleboro, USA) fitted with an 8 mm flat-end cylindrical plunger was used to determine the textural properties. The piston compressed the sample with a trigger force of 5 *g* until it reached 60% of the sample length at a pretest speed of 5 mm/s and a test speed of 2 mm/s. A shear fixture (TA-SBA-WB-l) was used to measure the shear force.

### 2.9. Muscle Liquid Holding Capacity (LHC), Cooking Loss, and pH

The LHC was measured using the gravimetric method [26], and the specific operation method is as follows: 1 g of boneless muscle (S) was wrapped in filter paper with a constant weight (V1) and placed into a 50 mL tube for centrifugation. Muscleless moist filtering paper (V2) was weighed after centrifugation and dried to constant weight (V3) in an oven at 75°C.

The method of Yun et al. [13] was used to assess muscle cooking loss. The muscle samples (M1) were placed in sealed heat-resistant plastic bags and then steamed for 5 min at 100°C. The muscle samples were weighed again (M2) after cooling.

Fresh muscle (1 g) was homogenized with 9 mL of distilled water. The pH of the muscle homogenates was measured using a pH analyzer (Leici, Shanghai, China).

### 2.10. Histomorphology Analysis

The preparation and staining of tissue sections were slightly modified in accordance with previous studies [25], and the specific steps were as follows: after washing with PBS, the fixed muscle blocks were trimmed into cuboids to discriminate between the transverse and longitudinal myofiber sections. After progressive dehydration with ethanol and transparent with xylene, muscle blocks were fixed in paraffin and serially sliced into 5 *μ*m thin pieces with a microtome (Zeiss, Oberkochen, Germany). Finally, the sections were stained using an HE staining kit (Solarbio, Beijing, China) and scanned using an optical microscope (Zeiss, Oberkochen, Germany). The diameter and number of muscle fibers were measured using ZEN 2 Lite software according to a previously described detection method [27].

### 2.11. Determination of Muscle Collagen Content

Total collagen content was calculated by multiplying the hydroxyproline (Hyp) content by 7.25 [28]. The hydroxyproline content was measured using a hydroxyproline kit (Nanjing Jiancheng, Nanjing, China) according to the manufacturer's instructions.

Heat-soluble collagen in muscle was evaluated using a previously described method [29]. Muscle samples were homogenized with Ringer's solution and heated at 77°C for 70 min. Next, the homogenate was centrifuged for 30 min at 4°C, and the supernatant was collected. This extraction was repeated twice. Finally, the heat-soluble collagen content of the supernatant was determined as described previously. The heat-insoluble collagen content was calculated by subtracting the heat-soluble collagen content from the total collagen content.

### 2.12. Determination of Relative Fatty Acid Content

The relative amounts of fatty acids in the feed and muscle were determined using a gas chromatography system (Agilent, New York, USA), as previously described [30].

### 2.13. Real-Time Polymerase Chain Reaction

Total RNA was isolated from the samples using the TRIzol reagent (Takara Bio, Japan). RNA concentration was determined using a spectrophotometer (Thermo, Massachusetts, USA), and RNA quality was determined using 1% agarose gel electrophoresis. HIScript II Reverse Transcriptase (Takara, Osaka, Japan) was used to reverse transcribe RNA after gDNA was erased. The expression of target genes was detected by diluted CDNA on a Light Cycler 480 instrument (Roche, Basel, Switzerland) using the ChamQ Universal SYBP qPCR Master Mix kit (Vazyme, Nanjing, China) according to the manufacturer's instructions. *18S* rRNA was used as the reference gene to standardize the target gene expression levels. All primers were validated, and the amplification efficiency was calculated. All primers had an amplification efficiency between 90% and 105%, as calculated using the following formula: *E* = 10 ^(−1/slope)^ −1.  Table 2 shows the sequences and amplification efficiencies of all the primers. The relative mRNA abundance of each gene was determined using the 2^−*ΔΔ*CT^ method.

### 2.14. Data Analysis

In this study, 225 common carp were used as the experimental animals. Three cages were assigned to each treatment, resulting in 45 fish per treatment group. Three fish were randomly selected from each cage for all data analyses, and nine replicates were used for each treatment group. Before analyzing the data, the Shapiro–Wilk and Levene tests were used to check the normality and variance homogeneity of all data. One-way analysis of variance was used for data with similar variances, and Duncan's multiple comparison test was used for post hoc tests when the difference was significant (*P* < 0.05). The nonparametric Mann–Whitney *U* test was applied when there was no homogeneous variance in the data, and pairwise multiple comparisons were used when the differences were significant (*P* < 0.05). All data are presented as the mean and standard error (SEM). SPSS (IBM, New York, USA) was used for statistical analysis, and GraphPad 6.02 (GraphPad Software, Massachusetts, USA) was used for graphical representation. Fatty acid correlation analysis was performed using the OmicStudio tools at https://www.omicstudio.cn.

## 3. Results

### 3.1. The Growth Performance

At the end of the experimental period, the OFO-fed fish had lower body weights than the fish in the FO groups (Table 3). Compared to the FO group, the final body weight (FBW), specific growth rate (SGR), weight gain rate (WGR), and feed efficiency (FE) were significantly reduced in the OFO group (*P* < 0.05). Compared with the OFO group, the FBW, SGR, WGR, and FE in the OT4 group were significantly higher (*P* < 0.05), but there was no significant difference between the OT4 and FO groups (*P* > 0.05). However, the FR and FCR trends were opposite to those of the above indicators. The survival rate of carp was 100% after the 70-day feeding trial. There were no significant differences between the groups in the condition factor (CF), viscerosomatic index (VSI), and hepatosomatic index (HSI) (*P* > 0.05). The optimal taurine supplementation level was 4 g/kg.

### 3.2. Antioxidant Enzyme Activities and Peroxide Levels

**Figure 1:**
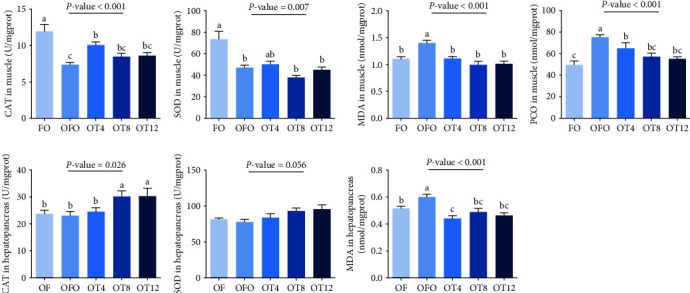
Antioxidant enzyme activity and peroxide levels in muscle and hepatopancreas of the common carp (*C. carpio* L.). Data in the figures are mean, and standard errors are represented by vertical bars (*n* = 9). Different symbols denote significant differences (*P* < 0.05).

We measured the concentration of MDA in the muscle and hepatopancreas, as well as the muscle protein carbonyl content, to confirm oxidative stress in fish following OFO ingestion. As illustrated in Figure 1, fish fed the OFO diet exhibited increased MDA levels in the hepatopancreas and muscles (*P* < 0.05), although both parameters significantly decreased after taurine administration (*P* < 0.05). The PCO in the muscle showed the same trend as that of MDA (*P* < 0.05). Compared to fish in the FO group, OFO-fed fish showed lower CAT and SOD activities in the muscle (*P* < 0.05) but no significant difference in CAT and SOD activities in the hepatopancreas (*P* > 0.05). Compared to the OFO group, taurine supplementation did not change SOD activity in the muscle or hepatopancreas, but supplementation with 4 g/kg taurine increased CAT enzyme activity in the muscle (*P* < 0.05), whereas supplementation with 8–12 g/kg taurine increased CAT enzyme activity in the hepatopancreas (*P* < 0.05).

### 3.3. Muscle Composition and Physicochemical Properties

As illustrated in Table 4, fish fed the OFO diet exhibited decreased muscle pH, lightness, and crude lipid levels (*P* < 0.05), although these three parameters significantly increased after 4–12 g/kg taurine administration (*P* < 0.05). Liquid loss and water loss were significantly decreased with 4–12 g/kg taurine supplementation compared to that of the OFO group (*P* < 0.05).

### 3.4. Muscle Texture Profile

**Figure 2:**
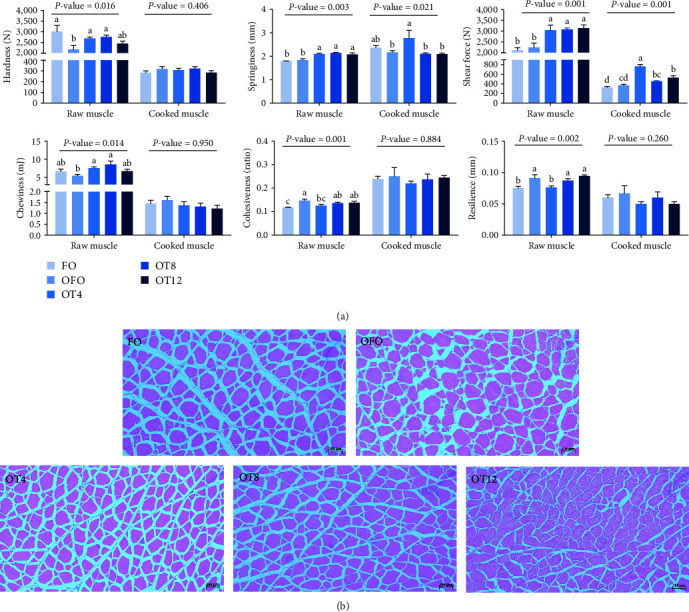
The muscle texture properties and histological sections of the common carp (*C. carpio* L.): (a) the raw and cooked muscle texture in the common carp (*n* = 9); (b) histological sections of cross-sectional myofibers of white myofibers on the back of the common carp were examined by H&E (*n* = 3). Different symbols denote significant differences (*P* < 0.05).

**Figure 3:**
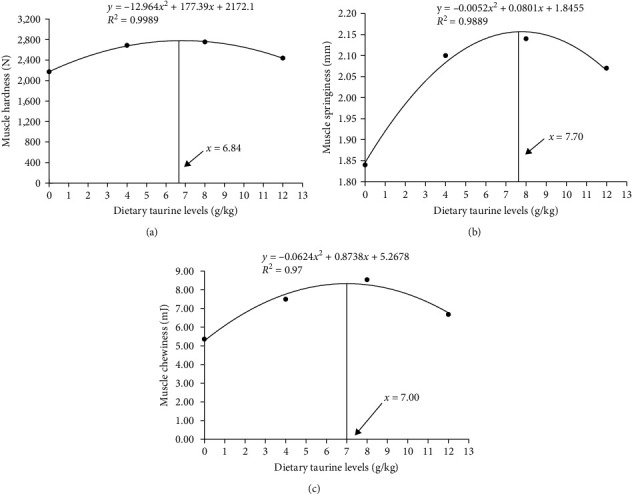
Quadratic regression analysis of muscle hardness (a), muscle springiness (b), and muscle chewiness (c) fed with graded dietary taurine (0, 4, 8, or 12 g/kg). Data are represented as mean.

As shown in Figure 2(a), dietary oxidized fish oil and taurine had a greater influence on raw muscle than on cooked muscle. Compared to the FO group, the hardness of raw muscle in the OFO group was significantly lower (*P* < 0.05), although cohesiveness and resilience were considerably higher (*P* < 0.05). It is worth mentioning that taurine supplementation considerably enhanced the hardness, shear force, chewiness, and springiness of raw muscle (*P* < 0.05). Cooked muscle springiness and shear force in the OT4 group were substantially higher than in the other groups (*P* < 0.05). The optimal taurine supplementation levels were 6.84, 7.70, and 7.00 g/kg according to the quadratic regression analysis of hardness (Figure 3(a)), springiness (Figure 3(b)), and chewiness (Figure 3(c)), respectively.

### 3.5. Muscle Histomorphology

As shown in Figure 2(b), OFO supplementation resulted in a loose and irregular arrangement of myofibers, a widening of myofiber gaps, and a tendency for gradual decomposition of the entire muscle tissue. The results of the statistical analysis of myofiber diameter and density are shown in Table 5. Myofiber density in the OFO group was lower than that in the FO group (*P* > 0.05), whereas myofiber diameter showed the opposite trend (*P* > 0.05). Compared with the OFO group, dietary supplementation with 4–8 g/kg taurine reduced myofiber diameter (*P* < 0.05) and increased myofiber density (*P* < 0.05). Taurine supplementation increased the percentage of small-diameter myofibers (<25 *μ*m) and decreased the percentage of middle-diameter myofibers (25–50 *μ*m) in a dose-dependent manner compared with the OFO group (*P* < 0.05). Meanwhile, the taurine group had fewer large-diameter myofibers (>50 *μ*m) than the OFO group (*P* > 0.05).

### 3.6. Gene Expression

**Figure 4:**
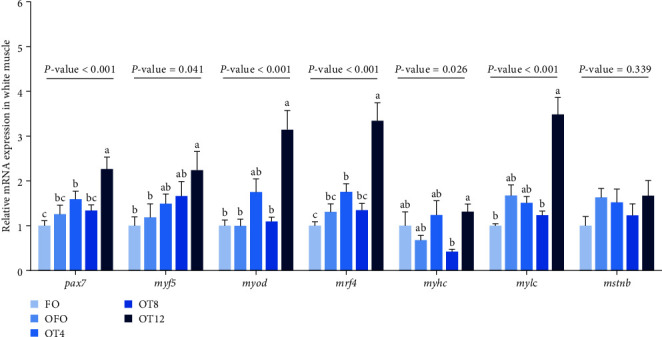
Effects on the expression of genes related to myofiber development in the muscle of the common carp (*C. carpio* L.) (*n* = 9). Different symbols denote significant differences (*P* < 0.05).

We evaluated the expression of muscle fiber development genes to gain further insight into the effect of taurine on muscle fiber development in OFO-treated fish.  Figure 4 illustrates that supplementation with 12 g/kg taurine increased the relative mRNA expression levels of paired box 7 (*pax7*), myogenic factor 5 (*myf5*), myogenic differentiation antigen (*myod*), and myogenic regulatory factor 4 (*mrf4*) in the muscle compared to those in the OFO group (*P* < 0.05). In addition, compared with the FO, the relative mRNA expression level of myostatin (*mstnb*) was increased in OFO (*P* > 0.05).

### 3.7. Muscle Collagen Content

Table 6 shows that the muscle collagen in each experimental group was mainly composed of heat-soluble collagen; however, the difference in heat-insoluble collagen was more significant. Compared with the OFO group, taurine supplementation did not change the content of heat-soluble collagen (*P* > 0.05) but increased the content of heat-insoluble collagen and total collagen in a dose-dependent manner (*P* < 0.05).

### 3.8. “Diet-Muscle” Fatty Acid Correlation

In total, 19 fatty acids were identified in the diet and muscles, as shown in Tables 7 and 8. Compared to the FO group, the muscle content of saturated fatty acids (SFAs) in the OFO group increased significantly (*P* < 0.05; Table 8), mostly palmitic acid (PA, C16 : 0). Compared to the FO group, fish fed the OFO diet exhibited a decreased n-3 polyunsaturated fatty acids (PUFA) content and n-3/n-6 PUFA ratio in the muscle (*P* < 0.05). Compared with the OFO group, the OT8 and OT12 groups had a significantly increased n-6 PUFA content in muscle (*P* < 0.05). However, the SFA, monounsaturated fatty acid (MUFA), and n-3 PUFA contents in the taurine groups were not significantly different from those in the OFO group (*P* > 0.05).

**Figure 5:**
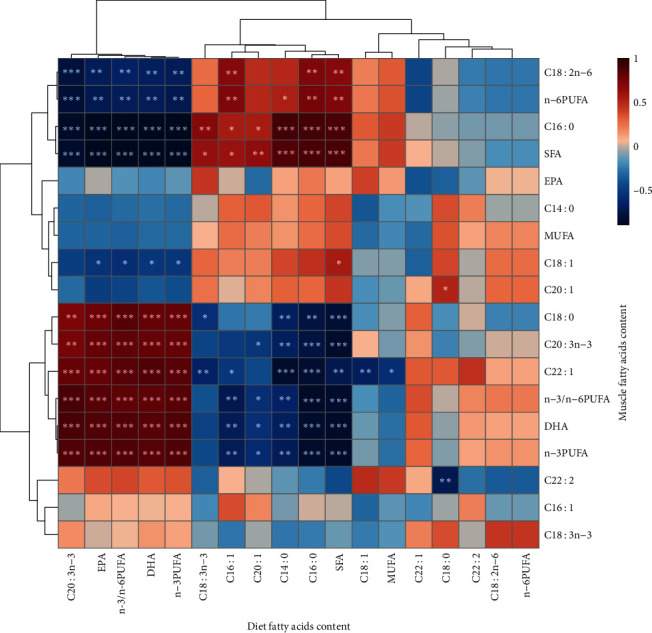
Heat map of the correlation between diet and muscle fatty acids of the common carp (*C. carpio* L.). The depth of the color in the figure represents the strength of the correlation; the darker the color, the stronger the correlation; otherwise, the opposite is true. Warm-colored lines represent positive correlations, and cool-colored lines represent negative correlations. The asterisks represent the significance of the correlation values ( ^*∗*^*P* < 0.05,  ^*∗∗*^*P* < 0.01, and  ^*∗∗∗*^*P* < 0.001).

The association between fatty acid content in the muscles and diet was investigated using Pearson's relationship analysis (Figure 5). The SFA content of the muscle was positively linked with the SFA content of the diet, particularly the myristic acid triglyceride (C14 : 0) and palmitic acid triglyceride (C16 : 0) (*P* < 0.001, Mantel's *r* ≥ 0.8). The corresponding components in muscle rose as dietary DHA, n-3 PUFA, and n-3/n-6 PUFA levels increased (*P* < 0.001, Mantel's *r* ≥ 0.8). Not surprisingly, the n-3/n-6 PUFA ratio in the diet was actively connected with the n-3/n-6 PUFA ratio and n-3 PUFAs content (mostly C20 : 3n-3, docosahexaenoic acid (DHA), and eicosapentaenoic acid (EPA) in muscles (*P* < 0.001, Mantel's *r* ≥ 0.8). Unlike DHA, there was a negative correlation between EPA levels in feed and muscle, although the correlation was not significant (Mantel's *r* = −0.02, *P*=0.94).

## 4. Discussion

The environment easily oxidizes unsaturated fatty acids in feed during processing, transportation, and storage. An oxidized lipid diet can cause oxidative stress in fish, negatively affecting their growth and muscle quality. This study showed that taurine effectively alleviated muscle damage caused by oxidized lipids in carp and showed an excellent ability to improve muscle quality, especially in terms of muscle growth, physicochemical properties, texture properties, collagen content, and antioxidant regulatory capacity.

### 4.1. Growth Performance and Muscle Nutrient Composition

Dietary OFO inhibited the growth performance of *C. carpio* L., which was in agreement with the research on hybrid grouper (♀*Epi-nephelus fuscoguttatus* × ♂*Epinephelus lanceolatus*) [17], and blunt snout bream (*M. amblycephala*) [6]. The main reason for this is the toxic chemicals (aldehydes, ketones, and acids) produced when oil is oxidized [17], which lower the nutritional content and palatability of the feed [33], resulting in oxidative stress and disrupting the physiological balance of fish [16]. In addition, hydroperoxides and alkyl radicals produced as a result of lipid oxidation continue to oxidize other lipids after entering the animal body [34]. As a result, fish have less energy accessible, leading to less energy deposition (in the present study, lower lipid content) and hence inferior growth performance. This is consistent with studies on farmed tilapia (*O. niloticus*), in which farmed tilapia fed an oxidized fish oil diet exhibited inhibited growth performance and reduced crude fat content [16]. In addition, a previous study has shown that high dietary n-3 HUFA levels increased lipid content in fish meat by activating lipid-sensitive transcription factors such as peroxisome proliferators-activated receptor gamma (PPAR*γ*) [35]. Therefore, the low n-3 PUFA levels in the feed in this study may have contributed to the low muscle lipid content in the OFO group.

Compared with the OFO group, supplementation with 4 g/kg taurine significantly improved the growth performance of carp, accompanied by the highest level of crude lipids in the muscle. This is consistent with the results obtained for turbot (*S. maximus* L.) [9]. Taurine has been shown to improve the growth performance of various fish species, such as golden pompano [8], Japanese flounder (*Paralichthys olivaceus*), and juvenile turbot (*S. maximus*) [36]. However, dietary taurine treatment had little effect on rainbow trout's (*Oncorhynchus mykiss*) growth performance [37]. The reason for this phenomenon may be that different fish have different growth requirements for taurine owing to their different taurine synthesis abilities. Studies have shown that carp (*C. carpio*) has a relatively stronger ability to synthesize taurine than Japanese flounder (*P. olivaceus*) [38]. Nonetheless, supplementation with 10–15 g/kg taurine in the feed still promoted the growth performance of juvenile carp [39]. These results indicate that although carp have strong taurine synthesis ability, this ability is still limited, and taurine needs to be obtained from the diet. Dietary taurine supplementation showed an effective way of counteracting the growth-inhibiting effects of oxidized lipid diets in common carp in the present study. It showed that an appropriate level of taurine (6.04 g/kg) added to an oxidized lipid diet was beneficial for carp weight gain.

### 4.2. Antioxygenic Property

The promoting effect of taurine on growth performance may be related to the antioxidant capacity of the fish. Therefore, the present study evaluated the effects of taurine on oxidative stress and antioxidant capacity in fish that were fed oxidized lipids. Oxidative stress occurs when the balance between the oxidative and antioxidant systems is disrupted, resulting in damage to DNA, lipids, and proteins [40]. PCO and MDA reflect the degree of peroxidative damage to proteins and lipids, respectively [41, 42]. SOD and CAT are important indices of antioxidant capacity and play vital roles in the balance between oxidation and peroxidation [43]. In this study, dietary oxidized lipids lowered muscle SOD and CAT activities, accompanied by high levels of oxidative stress in the muscle and hepatopancreas. In addition, this study found that taurine relieves peroxidation in muscle and hepatopancreas and increases antioxidant enzyme activity. Taurine has free radical scavenging activity (for example, hydrogen peroxide, superoxide, and peroxynitrite) and can reduce lipid peroxidation levels [7, 44]. In addition, taurine improves mitochondrial integrity and protects mitochondria from superoxide free radicals by preventing mitochondrial calcium overload and transfer of respiratory chain electrons [45]. This ultimately reduces reactive oxygen species produced by mitochondrial damage. As a result, taurine effectively improved the antioxidant properties of the common carp to alleviate the oxidative stress produced by dietary oxidized lipids. The antioxidant properties of muscles are strongly correlated with their physicochemical properties and color. Therefore, this study assessed the influence of taurine on the physicochemical properties of the common carp muscle.

### 4.3. Muscle Physicochemical Properties and Color

Muscle quality is significantly influenced by physicochemical characteristics, including cooking loss, liquid-holding capacity (LHC), and pH [25]. LHC comprises liquid loss, water loss, and lipid loss and indicates the capacity to stop lipids and water from escaping from muscle structures. Lower pH causes lower hardness and greater drip loss, both of which impair muscle quality [26]. Muscle pH is closely related to LHC because a high pH causes muscle proteins to carry more negative charges, which can enhance their ability to interact with water [46]. In this study, the muscle pH of carp was significantly reduced from 6.93 to 6.71 after being fed the oxidized lipid diet. However, taurine supplementation improved muscle LHC and pH compared to those in the OFO group. Studies have shown that fluctuations in muscle pH after slaughter are influenced by the accumulation of lactic acid during glycolysis [46]. Dietary oxidized fish oil causes oxidative stress, which promotes glycolysis to meet energy needs [16]. In addition, glycolysis involves both aerobic and anaerobic pathways, the former producing carbon dioxide and the latter lactic acid. Both products decreased the pH of the tissues. In this study, the muscle lactate content did not differ between the FO and OFO groups, implying that the oxidized lipid diet may have promoted glycolytic aerobic respiration to reduce muscle pH. Taurine functions as a buffer component in the mitochondrial matrix to maintain a slightly alkaline pH gradient and normal mitochondrial function [47]. In conclusion, taurine can effectively alleviate low muscle pH caused by oxidized lipids and improve the water-holding capacity of the muscle, thereby improving muscle quality.

Consumers have expectations regarding muscle color and typically use it as a sign to make purchasing decisions [48]. In this study, lightness (*L* ^*∗*^) substantially decreased in the dorsal muscle after feeding with an oxidized lipid diet. However, *L* ^*∗*^ values improved after taurine supplementation. The *L* ^*∗*^ value is one of the best indicators for measuring meat color strength and is frequently used as a foundation for product classification [49]. Free water increases as the pH approaches the isoelectric point of some water-bound muscle proteins, scattering more light and giving the tissue a brighter look [50]. According to a previous study, the glycolytic power of pig muscle increases as muscle color intensity increases [51]. Another study showed that higher glycolytic potential promotes acidity, which leads to lower *L* ^*∗*^ values [52]. In this study, the low pH of the OFO group may have moved away from the protein isoelectric points in the muscle, thereby reducing the presence of free water, inhibiting light reflection, and ultimately reducing the *L* ^*∗*^ of the muscle. In contrast, the addition of taurine increased muscle lightness via the opposite mechanism. In conclusion, muscle color change is closely related to glycolytic capacity and pH. However, further research is needed to determine the specific mechanism.

### 4.4. Myofiber Development

Fish myofibers are the basic units of muscle, and their density and diameter are frequently used to assess fish muscle quality [53]. Unlike other vertebrates, the muscles of teleosts grow via hypertrophy and hyperplasia [54]. In hypertrophy, the growth of pre-existing myofibers indicates an increase in diameter, whereas in hyperplasia, the formation of new myofibers increases the density of myofibers [55]. These processes are regulated by four myogenic regulators (MRFs): myogenic factor 5 (Myf5), myogenin (Myog), myogenic differentiation antigen (Myod), and myogenic regulatory factor 4 (Mrf4) [56]. Primary myogenic regulators (Myod and Myf5) are involved in myoblast fate determination and proliferation, whereas secondary myogenic regulators (Myog and Mrf4) regulate myoblast differentiation and fusion to form myofibers [57]. The paired homeobox gene Pax7 is responsible for most somatogenic processes required for muscle satellite cell development and regeneration [54]. Myosin is composed of two myosin heavy chains (Myhc) and two myosin light chains (Mylc), and two hexamers that regulate the light chain composition are essential for proper myocyte function [58]. In this study, taurine supplementation increased the expression levels of *pax7*, *myf5*, *myod*, and *mrf4*, accompanied by smaller-diameter myofibers and greater-density myofibers, compared to the OFO group. This indicated that taurine increases the expression levels of MRF family genes to stimulate hyperplastic myofiber formation. This is similar to the results of a study on juvenile turbot (*S. maximus*), in which the addition of taurine increased the number of hyperplastic myofibers [36]. In addition, myostatin (Mstn) is a negative muscle growth factor that inhibits muscle development by restraining the differentiation and proliferation of myogenic progenitor cells [59]. In this study, dietary OFO enhanced the expression level of *mstnb*, which may be the main reason for the decreased myofiber density.

### 4.5. Muscle Texture

The textural characteristics of fish are strongly correlated with the diameter and density of myofibers [36]. Therefore, this study analyzed the textural characteristics of the common carp muscle. The textural properties of fish muscles are important indicators of muscle quality, including hardness, springiness, chewiness, shear force, resilience, and cohesiveness [60]. In general, increasing the hardness of fish muscle improves fish quality [61]. Muscle hardness also improved with an increase in myofiber density [36]. Muscles with a hard texture have thin myofibers, whereas those with a soft texture have thick myofibers [53]. In the present study, dietary OFO considerably reduced muscle hardness, whereas hardness, shear force, chewiness, and springiness significantly improved after taurine treatment. This phenomenon was closely related to the small diameter and high density of myofibers induced by taurine treatment. It has also been shown that collagen provides high shear strength, making trout's (*Salmo irideus*) meat stiffer [62]. Therefore, collagen content in the muscle was examined, and it was discovered that collagen content increased with taurine supplementation in the diet. In this study, the contribution of taurine to hardness was closely related to small-diameter and large-density myofibers and high collagen content in the muscle.

### 4.6. “Diet-Muscle” Fatty Acid Correlation

This study revealed that the fatty acid content of muscle was affected by diet composition. Another study found that when Lagowski's minnow (*Rhynchocypris lagowski* Dybowski) was fed an oxidized fish oil diet, the fatty acid content of the tissues (muscle and hepatopancreas) was linearly related to the diet [63]. Studies on juvenile yellow drums (*Nibea albiflora*) have shown that the corresponding composition of muscle increased with an increase in DHA, EPA, n-6 PUFAs, and n-3 PUFAs in the diet [64]. However, unlike the performance of DHA, EPA in the muscle did not correlate strongly with diet in our study. Previous studies have demonstrated that both low- and high-EPA diets can have high DHA transfer rates and low EPA transfer rates from feed to muscle [65, 66]. Preferential EPA catabolism and DHA retention have also been found in Atlantic salmon (*Salmo salar* L.) and rainbow trout (*O. mykiss*) [67, 68]. Therefore, this phenomenon may be caused by the low retention of EPA in the muscles.

PUFAs are susceptible to peroxidative damage upon exposure to light, oxygen, or high temperatures. Because of the location of the double bonds in PUFAs, n-3 PUFAs are more vulnerable to lipid oxidation. In this study, the OFO diet increased the SFA contents while decreasing the n-3 PUFA content and n-3/n-6 PUFA ratio compared to the FO diet. Similar results were obtained in the rice field eel (*M. albus*) and channel catfish (*Ictalurus punctatus*) [5, 69]. The n-3 PUFAs, particularly DHA and EPA, have been shown to protect against various disorders, including cardiovascular diseases, inflammatory diseases, insulin resistance, and prostate cancer [70]. Furthermore, n-3/n-6 PUFA has been shown to play an essential role in regulating and controlling developmental performance, lipid metabolism, bone development, and muscle quality [5, 64]. This indicated that the oxidized oil diet reduced the nutritional value of the muscles. To explore further, we focused on fatty acid storage and usage in tissues, such as *β*-oxidation and fatty acid desaturation. Fat in fish supplies energy for fundamental life activities, mostly through the *β*-oxidation of fatty acids. It was found that muscle *β*-oxidation preferentially oxidized PUFA in Atlantic salmon (*S. salar*) [71]. In addition, an oxidized fish oil diet has been reported to increase SFA contents in tissues by preventing fatty acid desaturation in rats [72]. Therefore, the OFO diet in this study may influence muscle fatty acid composition by promoting *β*-oxidation of PUFAs and inhibiting desaturation of fatty acids. However, in the present study, taurine could increase the content of n-6 PUFA in muscle. This is different from a study on rice field eel (*M. albus*), where taurine increased n-3/n-6 PUFA in muscle and improved the nutritional value of the muscle [5]. In another study, the addition of taurine to the normal diet did not affect the fatty acid profile of juvenile California yellowtail (*Seriola dorsalis*) [73]. This may be due to differences in fish species and the nutritional composition of the diet.

## 5. Conclusion

In summary, this study showed that lipid oxidation-fed carp exhibited multiple negative effects, including growth inhibition and decreased muscle pH, brightness, hardness, crude lipid content, n-3/n-6 PUFA ratio, and muscle fiber density. It also increased the MDA and PCO levels in the muscles of the common carp. However, taurine supplementation significantly increased the content of n-6 PUFA in muscle. Moreover, taurine supplementation alleviated the other negative effects caused by lipid oxidation. Specifically, supplementation with 4 g/kg taurine significantly alleviated growth inhibition in common carp. On the whole, 6.84–7.70 g/kg taurine supplementation in an oxidized lipid diet improved muscle quality by improving muscle antioxidant defense ability, enhancing muscle physicochemical properties, promoting muscle fiber hyperplasia and collagen contents, and improving muscle texture properties. Overall, these results suggest that dietary taurine supplementation is an effective strategy for mitigating the deleterious effects of dietary lipid oxidation and for improving muscle quality in common carp.

## Figures and Tables

**Table 1:** Ingredients composition and nutrient content of experimental diets.

Items (g/kg, unless noted)	Groups				
	FO	OFO	OT4	OT8	OT12
Fish meal^a^	200.00	200.00	200.00	200.00	200.00
Cottonseed meal^b^	100.00	100.00	100.00	100.00	100.00
Soybean meal^c^	140.00	140.00	140.00	140.00	140.00
Rapeseed meal^d^	180.00	180.00	180.00	180.00	180.00
Wheat meal^e^	200.00	200.00	200.00	200.00	200.00
Fresh fish oil^f^	30.00	0.00	0.00	0.00	0.00
Oxidized fish oil	0.00	30.00	30.00	30.00	30.00
Ca (H_2_PO_4_)_2_^g^	25.00	25.00	25.00	25.00	25.00
Vitamins mix^h^	0.70	0.70	0.70	0.70	0.70
Minerals mix^i^	1.00	1.00	1.00	1.00	1.00
Lysine^j^	5.00	5.00	5.00	5.00	5.00
Microcrystalline cellulose^k^	118.30	118.30	114.30	110.30	106.30
Taurine^l^	0.00	0.00	4.00	8.00	12.00
Total	1,000.00	1,000.00	1,000.00	1,000.00	1,000.00
Proximate composition					
Crude protein	388.80	385.70	384.20	387.20	381.00
Crude lipid	85.03	86.53	89.35	88.77	87.45
Crude ash	93.00	93.20	91.30	92.90	91.60
Crude fiber	12.23	12.05	11.66	12.00	11.50
Moisture	85.70	88.60	89.30	86.40	89.70
Taurine^m^	1.06	1.04	5.04	9.24	13.37
TBARS ^*∗*^ (in diets; mg/kg)	4.22^b^	4.95^a^	4.88^a^	4.90^a^	4.82^a^

^a^Provided by Henan Tongwei Co., Ltd., Tangshan, China. 64% crude protein. ^b^Provided by Henan Tongwei Co., Ltd., Tangshan, China. 47% crude protein. ^c^Provided by Henan Hefeng Co., Ltd., Tangshan, China. 44.2% crude protein. ^d^Provided by Henan Tongwei Co., Ltd., Xinxiang, China. 35.7% crude protein. ^e^Provided by Henan Wudeli Co., Ltd., 13.4% crude protein. ^f^Provided by Dongxiang Chemical Co., Ltd., Xinxiang, China. ^g^Provided by Henan Tongwei Co., Ltd., Xinxiang, China. ^h^Provided by Zhuhai Weinuo Breeding Co., Ltd., Zhuhai, China. Vitamin premix (g/kg premix): Retinyl acetate, 0.66–1.98; cholecalciferol, 0.33–0.66; tocopherol, 9.00–30.00; menadione, 2.70–9.00; thiamine, 0.55–1.80; riboflavin, 0.86–2.88; cyanocobalamin, 0.01–0.02; nicotinamide, 10.00–42.00; folic acid, 0.90–3.00; pantothenic acid, 5.50–18.00; biotin, 0.02–0.06; ascorbicacid, 6.00–20.00; inositol, 7.20–24.00. ^i^Provided by Zhuhai Weinuo Breeding Co., Ltd., Zhuhai, China. Mineral premix (g/kg premix): Copper, 3.00–12.00; iron, 5.00–20.00; zinc, 8.00–32.00; manganese, 10.00–40.00; iodine, 0.20–0.80; selenium, 0.10–0.40. ^j^Provided by Beijing Solarbio Science and Technology Co., Ltd. ^k^Provided by Henan Tongwei Co., Ltd., Xinxiang, China. ^l^Shanghai Aladdin Biochemical Technology Co., Ltd. ^m^The value of taurine was measured by the method of high-performance liquid chromatography (HPLC). The method of HPLC was based on GB/T 5009.169–2003 with some minor modifications.  ^*∗*^TBARS, thiobarbituric acid-reactive substances.

**Table 2:** Real-time PCR primer sequences.

Gene	Sequence (5′–3′)	Tm (°C)	Efficiency (%)	GenBank ID	References
*pax7* ^a^	F: GCTCCATTAGTCGGGTTC R: GGCTCCGACTCCACATC	54.81 56.64	94.4	XM_042766854.1	[13]
*myf5* ^b^	F: GAGCCGCCACTATGAG R: TGGGAAGACGCTGACT	53.56 54.04	91.8	XM_019092315.2	[30]
*myod* ^c^	F: CAACGACACGCCAAAT R: CTGACAGCACGGGACA	52.59 55.77	97.8	XM_019068329.2	[31]
*mrf4* ^d^	F: ATGATGGACCTGTTTGAGAC R: TCACTTTTCTGAGATCTGGT	55.05 53.59	93	XM_019074759.2	[31]
*mylc* ^e^	F: ACAGAACCCAACCAACA R: GAATACACGCAGACCCT	52.97 53.26	97.2	XM_042731729.1	[25]
*myhc* ^f^	F: TGAACCCTCTGTGCTGT R: CTCCATACGCTTCTTGC	54.97 52.53	99.8	XM_042724157.1	[13]
*mstnb* ^g^	F: AACTCCGACTCAAACAGG R: ATGGTCTCAGTGGTGGC	54.12 55.95	95.5	XM_042764169.1	[25]
*18s*	F: GAGACTCCGGCTTGCTAAAT R:CAGACCTGTTATTGCTCCATCT	57.68 57.85	94.1	FJ710826.1	[32]

^a^ *pax7*, paired box 7. ^b^*myf5*, myogenic factor 5. ^c^*myod*, myogenic differentiation antigen. ^d^*mrf4*, myogenic regulatory factor 4. ^e^*mylc*, myosin light chain. ^f^*myhc*, myosin heavy chain. ^g^*mstnb*, myostatin.

**Table 3:** Growth performance of the common carp (*C. carpio* L.).

Index	Groups					*P*-value
	FO	OFO	OT4	OT8	OT12	
IBW^a^ (g)	8.94 ± 0.01	8.91 ± 0.02	8.89 ± 0.02	8.92 ± 0.02	8.89 ± 0.03	0.616
FBW^b^ (g)	95.40 ± 0.90^a^	88.39 ± 0.82^b^	93.26 ± 1.52^a^	89.96 ± 0.11^b^	89.58 ± 0.72^b^	0.002
WGR^c^ (%)	967.56 ± 10.20^a^	891.86 ± 11.82^b^	949.83 ± 17.47^a^	908.50 ± 2.11^b^	907.41 ± 5.79^b^	0.003
SR^d^ (%)	100 ± 0.00	100 ± 0.00	100 ± 0.00	100 ± 0.00	100 ± 0.00	1.000
SGR^e^ (%/day)	3.38 ± 0.01^a^	3.28 ± 0.02^b^	3.36 ± 0.02^a^	3.30 ± 0.00^b^	3.30 ± 0.01^b^	0.003
FE^f^ (%)	83.56 ± 0.87^a^	76.80 ± 0.81^b^	81.53 ± 1.47^a^	78.31 ± 0.11^b^	77.97 ± 0.67^b^	0.002
FCR^g^ (%)	1.20 ± 0.01^b^	1.30 ± 0.01^a^	1.23 ± 0.02^b^	1.28 ± 0.00^a^	1.28 ± 0.02^a^	0.002
FR (% bw/ day)^h^	2.68 ± 0.02^b^	2.87 ± 0.02^a^	2.74 ± 0.04^b^	2.83 ± 0.01^a^	2.84 ± 0.02^a^	0.002
CF^i^ (g/cm^3^)	2.49 ± 0.05	2.71 ± 0.09	2.63 ± 0.07	2.61 ± 0.09	2.57 ± 0.06	0.348
VSI^j^ (%)	9.61 ± 0.57	9.90 ± 1.00	9.88 ± 0.73	10.09 ± 0.80	10.03 ± 1.10	0.862
HSI^k^ (%)	2.43 ± 0.19	2.10 ± 0.07	2.14 ± 0.15	2.09 ± 0.17	2.45 ± 0.14	0.104

^a^IBW, initial body weight; ^b^FBW, final body weight; ^c^WGR (weight gain rate, %) = 100 × (final body weight − initial body weight)/initial body weight; ^d^SR, (survival rate, %) = 100 × final number of fish/initial number of fish; ^e^SGR (specific growth rate, %/day) = 100 × [ln (final body weight) − ln (initial body weight)]/experiment days; ^f^FE (feed efficiency, %) = 100 × (final body weight − initial body weight)/food intake; ^g^FCR (feed conversion ratio) = average individual dry matter feed intake/average individual weight gain; ^h^FR (feeding rate, % body weight/day) = 100 × average feed intake/[(IBW + FBW) /2]/days; ^i^CF (condition factor) = 100 × body weight/body length^3^; ^j^VSI (viscerosomatic index, %) = 100 × viscera weight/fish body weight; ^k^HSI (hepatosomatic index, %) = 100 × wet hepatopancreas weight/fish body weight. ^a–h^Values are means ± SEM (*n* = 3); ^i–k^ Values are means ± SEM (*n* = 9). Values in the same row with different superscripts represent statistically significant difference (*P* < 0.05).

**Table 4:** Muscle composition, liquid holding capacity (LHC), cooking loss, pH value, lactic acid content, and muscle color of the common carp (*C. carpio*. L).

Index	Groups					*P*-value
	FO	OFO	OT4	OT8	OT12	
Composition						
Moisture (%)	79.24 ± 0.39	79.35 ± 0.24	77.75 ± 1.15	79.30 ± 0.13	78.77 ± 0.46	0.335
Crude lipid (% WM)	1.80 ± 0.15^a^	1.26 ± 0.19^b^	2.07 ± 0.20^a^	1.78 ± 0.12^a^	1.76 ± 0.08^a^	0.018
Crude protein (% WM)	17.29 ± 0.34	17.42 ± 0.34	17.43 ± 0.99	17.13 ± 0.12	17.86 ± 0.18	0.883
Crude ash (% WM)	1.82 ± 0.18	1.73 ± 0.11	2.03 ± 0.33	2.16 ± 0.35	1.87 ± 0.13	0.756
LHC						
Liquid loss^a^ (%)	20.94 ± 1.97^a^	19.35 ± 1.12^ab^	17.10 ± 0.64^bc^	16.28 ± 0.77^c^	16.62 ± 0.91^c^	0.001
Water loss^b^ (%)	19.97 ± 0.63^a^	18.31 ± 0.98^a^	16.12 ± 0.62^b^	15.37 ± 0.76^b^	15.75 ± 0.77^b^	<0.001
Lipid loss^c^ (%)	0.98 ± 0.92	1.01 ± 0.15	0.99 ± 0.05	0.85 ± 0.07	0.87 ± 0.17	0.831
Cooking loss^d^ (%)	9.12 ± 0.68	9.94 ± 0.57	8.72 ± 0.91	9.29 ± 0.44	8.27 ± 0.61	0.532
pH	6.93 ± 0.03^a^	6.71 ± 0.04^b^	6.94 ± 0.03^a^	6.95 ± 0.03^a^	6.86 ± 0.02^a^	<0.001
Lactic acid (mmol/g protein)	1.08 ± 0.10	1.08 ± 0.06	0.95 ± 0.08	0.96 ± 0.07	0.90 ± 0.07	0.223
Muscle color						
Lightness (*L* ^*∗*^)	46.34 ± 0.60^a^	44.18 ± 0.48^b^	46.48 ± 0.54^a^	46.24 ± 0.39^a^	46.70 ± 0.29^a^	0.007
Redness (*a* ^*∗*^)	−2.00 ± 0.23	−2.19 ± 0.17	−2.08 ± 0.27	−2.41 ± 0.97	−2.40 ± 0.12	0.559
Yellowness (*b* ^*∗*^)	3.84 ± 0.40	3.91 ± 0.43	3.24 ± 0.61	3.55 ± 0.48	3.64 ± 0.31	0.723

^a^Liquid loss = 100 × (V2–V1)/S; ^b^Water loss = 100 × (V2–V3)/S; ^c^Lipid loss = 100 × (V3–V1)/S; ^d^Cooking loss = 100 × (M1–M2)/M1 WM, wet muscle. Values are means ± SEM (*n* = 9). Values in the same row with different superscripts represent statistically significant difference (*P* < 0.05).

**Table 5:** The myofiber density, diameter, and diameter distribution of the common carp (*C. carpio*. L).

Index	Groups					*P*-value
	FO	OFO	OT4	OT8	OT12	
Diameter < 25 *μ*m (%)	29.89 ± 2.07^b^	28.14 ± 1.37^b^	30.61 ± 2.93^b^	36.02 ± 1.06^a^	37.62 ± 0.59^a^	0.003
25 < Diameter > 50 *μ*m (%)	41.34 ± 2.16^ab^	44.15 ± 0.86^a^	44.31 ± 2.08^a^	40.37 ± 1.96^ab^	37.09 ± 0.84^b^	0.033
Diameter > 50 *μ*m (%)	28.78 ± 1.45^a^	27.71 ± 0.98^ab^	25.08 ± 1.56^ab^	23.61 ± 1.81^b^	25.29 ± 0.86^ab^	0.086
Mean diameter (*μ*m)	38.91 ± 0.72^ab^	40.10 ± 0.65^a^	36.86 ± 1.08^bc^	35.95 ± 0.56^c^	36.70 ± 0.50^bc^	0.002
Density (fibers/mm^2^)	239.77 ± 8.21^bc^	217.11 ± 6.842^c^	317.54 ± 27.43^a^	269.44 ± 14.15^b^	319.59 ± 15.33^a^	<0.001

Values are means ± SEM (*n* = 3). Values in the same row with different superscripts represent statistically significant difference (*P* < 0.05).

**Table 6:** Hydroxyproline and collagen content in the muscle of the common carp (*C. carpio* L.).

Index (*μ*g/mg)	Groups					*P*-value
	FO	OFO	OT4	OT8	OT12	
Total Hyp	0.22 ± 0.02^c^	0.24 ± 0.01^c^	0.23 ± 0.01^c^	0.29 ± 0.01^b^	0.35 ± 0.03^a^	<0.001
Heat-soluble Hyp	0.17 ± 0.01	0.19 ± 0.02	0.18 ± 0.02	0.19 ± 0.01	0.17 ± 0.01	0.939
Heat-insoluble Hyp	0.05 ± 0.02^c^	0.06 ± 0.01^bc^	0.05 ± 0.01^c^	0.11 ± 0.02^b^	0.17 ± 0.02^a^	<0.001
Total collagen	1.59 ± 0.13^c^	1.76 ± 0.02^c^	1.65 ± 0.06^c^	2.12 ± 0.06^b^	2.52 ± 0.18^a^	<0.001
Heat-soluble collagen	1.23 ± 0.09	1.35 ± 0.05	1.27 ± 0.11	1.35 ± 0.10	1.26 ± 0.08	0.939
Heat-insoluble collagen	0.35 ± 0.15^c^	0.41 ± 0.05^bc^	0.38 ± 0.10^c^	0.77 ± 0.13^b^	1.26 ± 0.15^a^	<0.001

Values are means ± SEM (*n* = 9). Values in the same row with different superscripts represent statistically significant difference (*P* < 0.05).

**Table 7:** Fatty acid composition of the experimental diets for the common carp (*C. carpio*. L) (% of total fatty acid, dry matter).

Fatty acid (%)	Groups					*P*-value
	FO	OFO	OT4	OT8	OT12	
C12 : 0	0.11 ± 0.00^b^	0.12 ± 0.00^a^	0.12 ± 0.00^a^	0.12 ± 0.00^a^	0.12 ± 0.00^a^	0.045
C14 : 0	4.71 ± 0.04^b^	5.04 ± 0.09^a^	5.12 ± 0.02^a^	5.04 ± 0.02^a^	5.09 ± 0.02^a^	<0.001
C15 : 0	0.43 ± 0.00^c^	0.42 ± 0.00^b^	0.45 ± 0.00^a^	0.45 ± 0.00^a^	0.45 ± 0.00^a^	<0.001
C16 : 0	18.18 ± 0.34	20.91 ± 0.08	21.08 ± 0.09	21.08 ± 0.02	21.14 ± 0.04	0.070
C16 : 1	5.88 ± 0.05^c^	5.99 ± 0.05^c^	6.16 ± 0.02^b^	6.14 ± 0.03^b^	6.35 ± 0.03^a^	<0.001
C17 : 0	0.42 ± 0.00	0.41 ± 0.02	0.44 ± 0.01	0.45 ± 0.00	0.45 ± 0.01	0.171
C17 : 1	1.04 ± 0.13	0.91 ± 0.02	0.93 ± 0.02	0.92 ± 0.02	0.93 ± 0.02	0.551
C18 : 0	3.45 ± 0.31	3.41 ± 0.04	3.43 ± 0.05	3.48 ± 0.06	2.91 ± 0.49	0.524
C18 : 1	25.38 ± 0.10	25.30 ± 0.10	25.04 ± 0.08	25.32 ± 0.07	25.35 ± 0.41	0.504
C18 : 2n-6	17.56 ± 0.23	17.63 ± 0.08	17.44 ± 0.05	17.51 ± 0.05	17.28 ± 0.15	0.401
C18 : 3n-3	2.09 ± 0.02	2.16 ± 0.03	2.14 ± 0.01	2.15 ± 0.01	2.12 ± 0.00	0.070
C20 : 1	1.93 ± 0.03^b^	1.93 ± 0.04^b^	2.13 ± 0.03^a^	2.07 ± 0.02^a^	2.13 ± 0.03^a^	0.001
C20 : 3n-3	0.55 ± 0.01^a^	0.45 ± 0.01^b^	0.44 ± 0.00^b^	0.43 ± 0.00^b^	0.44 ± 0.01^b^	<0.001
C20 : 5n-3 (EPA)^a^	6.66 ± 0.09	5.78 ± 0.05	5.53 ± 0.02	5.35 ± 0.02	5.68 ± 0.04	0.060
C22 : 1	2.20 ± 0.03	2.07 ± 0.04	2.21 ± 0.03	2.18 ± 0.03	2.10 ± 0.06	0.135
C22 : 2	0.20 ± 0.02	0.20 ± 0.02	0.22 ± 0.03	0.17 ± 0.01	0.18 ± 0.04	0.681
C22 : 6n-3 (DHA)^b^	9.96 ± 0.03^a^	7.25 ± 0.14^b^	7.15 ± 0.09^c^	7.12 ± 0.04^d^	7.31 ± 0.13^bc^	<0.001
*Σ* SFA^c^	26.97 ± 0.61^b^	30.33 ± 0.09^a^	30.63 ± 0.12^a^	30.62 ± 0.06^a^	30.14 ± 0.45^a^	<0.001
*Σ* MUFA^d^	36.00 ± 0.27	36.20 ± 0.15	36.46 ± 0.10	36.64 ± 0.11	36.86 ± 0.51	0.256
*Σ* n-3 PUFA^e^	19.26 ± 0.12^a^	15.63 ± 0.15^b^	15.25 ± 0.11^cd^	15.05 ± 0.04^d^	15.54 ± 0.10^bc^	<0.001
*Σ* n-6 PUFA^f^	17.56 ± 0.23	17.63 ± 0.04	17.44 ± 0.05	17.51 ± 0.05	17.40 ± 0.16	0.401
n-3/n-6 PUFA	1.10 ± 0.01^a^	0.89 ± 0.01^b^	0.87 ± 0.01^b^	0.86 ± 0.01^b^	0.90 ± 0.01^b^	<0.001

^a^C20 : 5n-3 (EPA), eicosapentenoic acid; ^b^C22: 6n-3(DHA), docosahexaenoic acid; ^c^SFA, saturated fatty acids; ^d^MUFA, monounsaturated fatty acids; ^e^n-3 PUFA, omega 3 polyunsaturated fatty acids; ^f^n-6 PUFA, omega 6 polyunsaturated fatty acids. Values are means ± SEM (*n* = 3). Values in the same row with different superscripts represent statistically significant difference (*P* < 0.05).

**Table 8:** Fatty acid composition of the white muscle of the common carp (*C. carpio* L.) (% of total fatty acid, dry matter).

Fatty acid (%)	Groups					*P*-value
	FO	OFO	OT4	OT8	OT12	
C14 : 0	2.00 ± 0.05	1.92 ± 0.11	2.01 ± 0.06	1.77 ± 0.11	1.89 ± 0.08	0.424
C16 : 0	18.68 ± 0.38^c^	21.93 ± 1.05^a^	21.68 ± 0.12^ab^	21.62 ± 0.14^ab^	20.98 ± 0.35^b^	<0.001
C16 : 1	6.18 ± 0.14	5.94 ± 0.19	5.93 ± 0.11	5.71 ± 0.11	6.29 ± 0.13	0.056
C18 : 0	3.51 ± 0.15^a^	3.13 ± 0.10^b^	3.15 ± 0.07^b^	3.42 ± 0.18^ab^	3.54 ± 0.10^a^	0.013
C18 : 1	23.23 ± 0.26	24.20 ± 0.59	23.95 ± 0.36	23.24 ± 0.39	23.14 ± 22.50	0.212
C18 : 2n-6	11.32 ± 0.15^c^	11.63 ± 0.22^bc^	12.25 ± 0.23^ab^	12.55 ± 0.24^a^	12.77 ± 0.21^a^	<0.001
C18 : 3n-3	0.95 ± 0.04	0.86 ± 0.03	0.96 ± 0.07	0.91 ± 0.08	0.81 ± 0.04	0.344
C20 : 1	2.02 ± 0.22	2.06 ± 0.21	2.02 ± 0.03	2.01 ± 0.05	1.86 ± 0.04	0.256
C20 : 2	0.24 ± 0.01^b^	0.25 ± 0.01^b^	0.25 ± 0.01^b^	0.29 ± 0.01^a^	0.26 ± 0.01^b^	0.011
C20 : 3n-6	0.23 ± 0.01^b^	0.35 ± 0.01^a^	0.35 ± 0.01^a^	0.39 ± 0.02^a^	0.38 ± 0.00^a^	<0.001
C20 : 3n-3	1.52 ± 0.10^a^	1.32 ± 0.07^b^	1.32 ± 0.02^b^	1.42 ± 0.04^ab^	1.43 ± 0.04^ab^	0.018
C20 : 5n-3 (EPA)^a^	5.28 ± 0.08	5.28 ± 0.19	5.15 ± 0.05	5.37 ± 0.06	5.42 ± 0.04	0.318
C22 : 1	0.49 ± 0.02^a^	0.25 ± 0.01^b^	0.29 ± 0.01^b^	0.28 ± 0.01^b^	0.28 ± 0.02^b^	<0.001
C22 : 2	0.18 ± 0.02	0.16 ± 0.00	0.12 ± 0.01	0.11 ± 0.02	0.16 ± 0.03	0.158
C22 : 6n-3 (DHA)^b^	22.65 ± 0.46^a^	19.30 ± 0.85^b^	19.13 ± 0.46^b^	19.50 ± 0.53^b^	19.27 ± 0.52^b^	0.001
*Σ* SFA^c^	27.10 ± 0.16^b^	29.86 ± 0.54^a^	29.72 ± 0.10^a^	29.71 ± 0.17^a^	29.22 ± 0.21^a^	<0.001
*Σ* MUFA^d^	32.55 ± 0.46	33.03 ± 0.81	32.77 ± 0.35	31.77 ± 0.51	32.13 ± 0.44	0.491
*Σ* n-3 PUFA^e^	30.40 ± 0.47^a^	26.79 ± 1.05^b^	26.55 ± 0.44^b^	27.19 ± 0.55^b^	26.94 ± 0.57^b^	0.002
*Σ* n-6 PUFA^f^	11.55 ± 0.15^c^	11.98 ± 0.24^bc^	12.60 ± 0.23^b^	12.94 ± 0.23^a^	13.15 ± 0.22^a^	<0.001
n-3/n-6 PUFA	2.64 ± 0.07^a^	2.23 ± 0.06^b^	2.11 ± 0.07^b^	2.11 ± 0.07^b^	2.11 ± 0.08^b^	<0.001

^a^C20 : 5n-3 (EPA), eicosapentenoic acid; ^b^C22: 6n-3(DHA), docosahexaenoic acid; ^c^SFA, saturated fatty acids; ^d^MUFA, monounsaturated fatty acids; ^e^n-3 PUFA, omega 3 polyunsaturated fatty acids; ^f^n-6 PUFA, omega 6 polyunsaturated fatty acids. Values are means ± SEM (*n* = 9). Values in the same row with different superscripts represent statistically significant difference (*P* < 0.05).

## Data Availability

All data for this study are included in the article and were obtained from the corresponding author upon reasonable request.
